# “Havens of mercy”: health, medical research, and the governance of the movement of dogs in twentieth-century America

**DOI:** 10.1007/s40656-021-00478-4

**Published:** 2021-12-02

**Authors:** Robert G. W. Kirk, Edmund Ramsden

**Affiliations:** 1 Centre for the History of Science, Technology and Medicine (CHSTM), University of Manchester, Manchester, UK; 2 School of History, Queen Mary University of London, London, UK

**Keywords:** Biomedicine, Laboratory animal, Dog, Antivivisection, Humane, Health

## Abstract

This article argues that the movement of dogs from pounds to medical laboratories played a critically important role in debates over the use of animals in science and medicine in the United States in the twentieth century, not least by drawing the scientific community into every greater engagement with bureaucratic political governance. If we are to understand the unique characteristics of the American federal legislation that emerges in the 1960s, we need to understand the long and protracted debate over the use of pound animals at the local municipal and state level between antivivisectionists, humane activists, and scientific and medical researchers. We argue that the Laboratory Animal Care Act of 1966 reflects the slow evolution of a strategy that proved most successful in local conflicts, and which would characterize a “new humanitarianism”: not the regulation of experimental practices but of the care and transportation of the animals being provided to the laboratory. Our analysis is consistent with, and draws upon, scholarship which has established the productive power of public agencies and civil society on the periphery of the American state.

## Introduction

Giving evidence to the 1966 Californian State Fact Finding Commission on the release of unclaimed, impounded animals for use in scientific research, the prominent antivivisectionist Larry Andrews warned that the National Society of Medical Research (NSMR) had “no compunction at all about converting havens of mercy, built by the contributions of people who oppose cruelty—many of them antivivisectionists—into collection depots for the vivisection laboratories” (Stiern, 1966: p. 114). “Havens of mercy” were animal shelters, established by a variety of humane societies across the United States from the late nineteenth century. The Californian State Fact Finding Commission had been convened to gather evidence on the need for a state law compelling animal shelters, or, as the scientific community preferred to call them, the municipal or city pound, to make unclaimed animals available for medical research. In this wide ranging and vociferous conflict even the naming of the institutions mattered. From a civic perspective the purpose of these institutions was to control lost and feral animals in the urban environment. Yet, for those that ran them, animal shelters were private charitable institutions providing refuge for lost and unwanted animals. And for the scientific community the pound was a public institution that wasted an undervalued resource by euthanizing animals rather than making them available for medical research. By 1966 the longstanding question of how the nation should treat its ownerless animals had become a major public concern and one of the most difficult political issues to resolve.

Animal shelters were a nineteenth-century response to urbanization and the challenge it posed to domesticated animals (Wang, 2019: pp. 193–226). While the animal shelter was established to protect animals from suffering, whether from starvation or human cruelty, the moral values that shaped this work were diverse, reflecting their time and place. Prevention of suffering, for example, did not always equate to the preservation of life. Historically, a painless death was widely considered humane. Often existing as private philanthropic entities, animal shelters were located on the unstable fault line between public and private in American society. Accordingly, they existed within a variety of complex, frequently contested, relationships with government that set limits on their activities through various financial (and sometime infrastructural) interdependencies. Expectations were codified through a mixture of informal agreement and municipal, state or even federal law. In her study of rabies and animal control in nineteenth-century New York, Jessica Wang has shown how, consistent with critical scholarship on the development of the American state, the diversity of animal shelters challenges the narrative of an increasingly formalized, centralized, and hierarchical system of municipal, state, and federal authority across the twentieth century (Wang, 2019: pp. 195–196).^1^ Similarly, Irvine has argued that animal shelters illustrate how the presence of animals has shaped the development of legislation in urban America due to their critical position “in the intricate relationship between public policy and private morality” (Irvine, 2015: p. 98).

Managing the movement of animals has long been central to the control of human and animal disease (Woods, et. al. 2017). Indeed, the historical study of shelters has outlined their role helping to control zoonotic disease such as rabies (Pearson, 2017; Wang, 2019; Worboys & Pemberton, 2007). By removing feral animals from urban spaces, shelters eliminated the risk of their serving as vectors or reservoirs of disease and so contributed to public health. However, by working to rehome lost family pets, shelters also worked to prevent animal cruelty. Shelters, therefore, entangled civic responsibility with multiple forms of humanitarian concern. For this reason, municipal governments frequently, though not consistently, contracted local humane societies to manage municipal animal shelters. Humane societies, run as charitable endeavors, were keen to stabilize their revenue by providing a public service compatible with their humanitarian commitments. *Pound release*, often recast as *pound seizure* by opponents and framed as the conversion of “havens of mercy” into “depots for the vivisection laboratories” where animals moved from shelters to medical laboratories, ran counter to this accord. From the perspective of the biomedical sciences, unwanted animals were more than a public health menace as they could serve the public health if made available to medical research. Animal experimentation, however, was a difficult subject for the humane movement. In this article, we reconstruct how antivivisectionists attempted to use the threat of pound release to align the influential yet moderate humane movement with their cause. During the twentieth century the status of family pets within American Culture grew as “affection for companion animals became fully integrated into mass consumer culture” As (Jones, 2002 p. 115). Accordingly, to the extent that pound release could be framed as a threat to the family pet, it provided antivivisectionists with an effective means to intensify public and political concern about it and build support for restrictions on the movement of dogs and cats to laboratories. Within subsequent debates, the public shelter (or pound) became a critically important space in which the value and purpose of animals in modern society was contested. Different communities articulated competing representations of the dog, as a dangerous and disease-ridden pest, as a pet, companion, and family member, a victim of vivisection, or an ally or resource in the fight against disease, illustrating, as Philip Howell has argued, that the dog in modern society is arguably “the most liminal of all animals” (2019: p. 147).

Controversy around animal research had ebbed and flowed within the public imagination since the emergence of antivivisectionist critiques in the nineteenth century (Lederer, 1995; Rudacille, 2000). At the time that the Californian State Fact Finding Commission was exploring the need for a state pound release law, the subject had risen to unprecedented nationwide attention. In 1966 the United States Congress passed what is generally understood to be the first federal legislation for the protection of laboratory animals. However, what was known as the Laboratory Animal Welfare Act was motivated more by the question of the place of domestic animals, particularly dogs, in American society than any intention to intervene in the practice of animal experimentation. With the nation engaged in the Vietnam war and at the height of the civil rights struggle, United States congressmen reported receiving more correspondence concerning the use of dogs in medical research than any other subject (Phillips & Bellotti, 2017; Stevens, 1968). Given this historical context, it would be reasonable to assume that Californian interest in pound release was predominantly shaped by the national debate. After all, Larry Andrews attended from Arizona, appeared on behalf of the National Humanitarian League, and he spoke against the NSMR. To assume this, however, would simplify a complex historical narrative and obscure an overlooked feature of what would become Public Law 98–544 or the Laboratory Animal Welfare Act 1966 (widely thought to be the first federal law to regulate animal research). This was the fact that it established no direct regulatory oversight over the experimental use of animals within scientific research.^2^ Instead, the Laboratory Animal Welfare Act aimed to “protect the owners of dogs and cats from theft of such pets, to prevent the sale or use of dogs and cats which have been stolen, and to insure that certain animals intended for use in research facilities are provided humane care and treatment” (US Public Law 89–544, 1966). The law only addressed laboratory animals indirectly, in that it regulated the sourcing, transport, and husbandry of animals destined for use in the laboratory. Why, we might ask, did a law that has been widely considered to have established regulations to protect laboratory animals in scientific research focus instead on the welfare of animals prior to their use in science?

To answer this question, we draw upon recent political historiography that recasts American state power as dispersed and horizontally organized, rather than vertically consolidated. In pursuing a more “pragmatic approach” focused on the “state in action rather than in theory”, William Novak has challenged the idea of clear distinctions between public and private spheres and instead sees their relationship as marked by ambiguity, hybridity, and “interpenetration… the convergence of public and private authority in everyday policymaking” (Novak, 2008: pp. 767, 770). State power is “widely distributed among an exceedingly complex welter of institutions, jurisdictions, branches, offices, programs, rules, customs, laws, and regulations” which, as Wang has observed, helps explain how animal shelters became entangled within municipal approaches to animal control (Novak, 2008: p. 765; Wang, 2019: p. 195). In this article, we build upon these observations to show how local political constellations became central mechanisms for contesting and negotiating the scope and limits of public health, biomedical research, and the activities of the life sciences. We show how the Laboratory Animal Welfare Act (1966) was the culmination of diverse, longstanding, protracted, and always contested settlements negotiated in city halls and state legislatures across the nation. Across these otherwise diverse debates, public concern was focused not on what happened to animals within biomedical research, but rather which animals were made available and how they would travel to the laboratory. Following Novak’s observation that “looking simply at the national center or the federal bureaucracy is to miss where much of the action is”, we argue that the public and political concern addressed by the Laboratory Animal Welfare Act (1966) was predominantly framed at the local and state levels, what Novak describes as the “periphery” (2008: p. 766), and as a result was focused on the question of how animals were sourced for biomedical research as opposed to their experience within the experimental laboratory.

## Pound release or pound seizure? Animal protectionism, animal control and the life sciences in California

As the United States mobilized its forces in 1917, the *National Humane Review* reported on the “war at home” describing “several bitter conflicts and hard fought battles carried on by humanitarians in the different states in order to protect animals” (Stillman, 1917b: p. 110). Published by the American Humane Association (AHA), the *National Humane Review* was intended to unite progressive animal protectionist societies from across the United States. The war that concerned animal protectionists was occurring on the streets of towns and cities across America. It involved access to animals, principally ownerless dogs, and it threatened to draw the humane movement into the bitter struggle between antivivisectionists and medical communities over the use of animals for experimental research.

In contrast to previous conflicts, where antivivisectionists sought to sway public opinion against animal experimentation to secure legal restrictions on the practice, the events of 1917 were triggered by members of the scientific community choosing to go on the offensive. In California, George H. Whipple, Director of The George Williams Hooper Foundation for Medical Research, declared his support for a state law that would make “available for laboratory purposes such unclaimed dogs and cats in the city pounds as otherwise will be destroyed” (Whipple, 1917: p. 68). Whipple explained that the study of the living animal body was critical to the life sciences and the national health. Furthermore, animals were always treated humanely. What was novel was the economic argument that unwanted impounded animals were a wasted national resource. 4000 dogs were killed annually in San Francisco’s animal shelters, yet a large research laboratory would use less than 200 a year. As the breeding of dogs was costly, researchers were dependent upon commercial dealers who were often “irresponsible persons who collect strays and sell them to the laboratory” (Whipple, 1917: p. 70). Whipple acknowledged the current risk that stolen pets may unknowingly be purchased by laboratories. Such an event could lead to “unpleasant complications” allowing “rabid antivivisectionists” to “spread misinformation before the public” (Whipple, 1917: p. 70). As such, allowing laboratories to establish a “rational agreement with the neighboring dog pound” would benefit science, society, and the national health.

This 1917 attempt to secure state pound release legislation for California was one prominent example of a renewed phase in the animal experimentation debate. Similar bills had been introduced the same year in Pennsylvania and New Jersey (Stillman, 1917b: p. 110). While the practical aim of securing access to large numbers of animals was important to scientific research, the process was equally significant. Mobilizing the scientific community behind permissive legislation provided an opportunity to “educate the public and to inform the state legislature concerning medical science” whilst shifting the tone of the legal debate against antivivisectionist “pernicious medical legislation” that sought (Whipple, 1917: p. 69). Whilst antivivisectionist campaigns to restrict or abolish animal experimentation were routinely unsuccessful, they were relentless and posed a risk of gradually eroding public support for medical science. In 1915, to counter this threat, Californian scientists established the California Society for the Promotion of Medical Research (CSPMR) and set out to appropriate the antivivisectionist legislative strategy for their own ends whilst simultaneously securing a reliable and affordable supply of animals.^3^ Pound release proved highly contentious however, in large part because proposed laws were vague as to whether they were targeting animal shelters, municipal pounds, or both. These categories were, in themselves, difficult to determine with any certainty at the time due to the considerable variability of practices of animal control across localities which reflected the wider “messy distribution of governmental authority in the United States” (Wang, 2019: p. 217).^4^ Some municipalities managed animal control through wholly owned public institutions commonly known as the city pound. Others outsourced this work to humane societies who ran the pound as a private animal shelter operating by contract to the city. Wang presents the varied development of the animal shelter as a further illustration of Novak’s thesis that “the particular genius of the American state may lie in its multilayered, decentralized, infinitely divisible character, as opposed to the Weberian stereotype of expert-led, rationalized bureaucracy as the essence of the modern state” (Wang, 2019: p. 194–195). Mid-twentieth century pound release campaigns were, however, an example of a rationalized and, crucially for the biomedical sciences, scientized bureaucracy. As such, the controversy around pound release legislation involved more than moral concern over the place of animals within society. It was equally shaped by the conflict between emergent rationalized bureaucracy and the decentralized and multifaceted model of governmental authority in America.

The 1917 Californian pound release campaign responded to what the scientific community identified as an overly complex, inconsistent, and irrational approach to animal control embodied in the institution of the animal shelter. Whipple reframed the ownerless dog as a (currently wasted) public resource of value to scientific research and the national health. Harnessing this resource required an efficient and rational approach to animal control in the modern urban environment. From the perspective of municipal government, animal control was a matter of civic order and the protection of public health. Once impounded, the fate of a stray dog mattered little. However, for the various animal protectionist and humanitarian societies operating animal shelters on behalf of municipal government, their work embodied a humanitarian commitment, often religiously motivated, of kindness toward lesser forms of life (Beers, 2006; Sperling, 1988). These differing sensibilities account for the differing terminologies. When, for instance, the Women’s Pennsylvania Society for the Prevention of Cruelty to Animals took on the responsibility for the Philadelphia public pound, it was renamed a “shelter” to convey its status as a merciful refuge for animals (Unti, 2002: pp. 167–173). Unlike the pound, the animal shelter primarily existed for the good of animals working to rescue and return animals to their home or bring their suffering to an end through a humane death. For humanitarians, all dogs were deserving of societal protection and of being considered neither public nor entirely private property. “There should be no discrimination against dogs or cats which are not born into homes of affluence”, argued Mrs Dell Hawksford, the Executive Secretary of the California Animal Defense and Anti-Vivisection League, as there were “provisions for unfortunates of all classifications—why not for defenseless dumb friends?” (Anon, 1938a). As intelligent, emotionally developed and loyal human companions, dogs should not become a problem to be disposed of simply because they were detached from a human owner. Rather, they should be treated on the model of “juveniles or other humans”, as akin to “wards of the city or county” making the animal shelter the legal guardian in the absence of an owner (Anon, 1938a). Widely shared amongst humanitarians, this extra-legal understanding of the dog as a ward conflicted with the logic of pound *release*. Indeed, humanitarians refused to use the term “release” for any such proposal, insisting on seeing it as *seizure*. The State Humane Association of California warned against forced movement of animals from shelters to laboratories, its secretary declaring that the 1917 bill would “nullify the principal relief work of many societies” by compelling shelters to act as “agents of the medical colleges … gathering up animals to be experimented upon” (Anon, 1917a). Rather than nullifying antivivisectionist critique, the campaign for pound release instead invigorated it by causing moderate humanitarians and animal protectionists to form uncomfortable alliances with the cause. One veteran Sacramento lobbyist reported their surprise that:

Medical men have displayed such poor judgment in their methods of legislation. They might have known that, if they fathered a bill providing for vivisection for stray cats and dogs, they would waken a storm of protest. Here is the diplomatic way to have handled that bill. The doctors should have kept mum about vivisection and should have hired someone to go to Sacramento and secure the passage of a bill which would give to the pound-keepers the privilege to sell or give away unredeemed animals in place of chloroforming them [making] the argument that the bill was in the interests of humane treatment of dumb animals; that stray children are not chloroformed, but that we try to find good homes for them and that the animals should be treated the same. He would have had all those humane societies back of him, and the bill would have passed almost unanimously. No one would have thought of vivisection. After it was a law the medical schools could have quietly made their terms with the pound men. (Anon, 1917b).

Instead, humane and animal protectionist societies across California united with antivivisectionists in opposition to the new threat of pound seizure. Presenting the attack upon the shelter as an attack upon lost pets allowed critics to sway political and public opinion against pound seizure. Without trust in the animal shelter, the public would be dissuaded from collecting and delivering animals if they knew they were destined for the laboratory (Anon, 1917a). The *Los Angeles Times* concluded that pound release risked lost pets being “hustled immediately to some college… Every time a pet is lost and never found the owner may wonder whether it was not stretched on a board while a surgeon gouged its living vitals” (Anon, 1917c). Such vivid language outpaced the scientific argument that the current system permitted nefarious dog-nappers to pose as respectable dog-breeders and sell stolen animals to unwitting laboratories.

With such widely shared disapproval, the 1917 Californian pound release bill was defeated (Anon, 1921: p. 75). Whipple and his allies quietly moved on, glad to escape what had become an embarrassing public controversy, but they did so without abandoning their belief that pound release was a public good. The mismanaged political campaign, however, had not only set the terms of the debate but gifted a new approach to antivivisectionism. Antivivisectionists, for so long the poor relation within animal advocacy, avoided by moderate animal protectionism, ignored by politicians, and reviled by the scientific community, discovered that the threat of pound seizure inspired alliances and brought popular support that their longstanding criticism of animal experimentation had so far failed to achieve. Events in California were widely reported within the antivivisectionist press, offering the movement a new credibility and a rare opportunity to celebrate that “anti-vivisection forces were victorious, in spite of the most determined and powerful influences our opponents could command from the legal, political, educational, state and medical authorities” (Anon, 1921: p. 75).

## Antivivisectionism by another name: the fight against pound seizure

American animal protectionism developed in urban areas, largely in response to local issues, driving the establishment of humanitarian societies in towns and cities which, in turn, formed state-wide bodies to represent the collective movement (McCrea, 1910; Shultz, 1924). In 1908, for example, the State Humane Association of California was established, growing to include fifty societies across the state by 1917 and boasting an individual membership of nearly 8000. Nationally, the humane movement was led by the American Humane Association (AHA), representing a broad community committed to the prevention of suffering. Among the diverse values, commitments, and concerns that motivated humanitarian societies, one particularly contentious issue was generally avoided: vivisection. In 1892 the AHA committed to a position on vivisection that attempted to satisfy members who were persuaded by antivivisection, as well as medically qualified humanitarians who warned against opposing scientific medicine as it did so much to relieve human and animal suffering. American humanitarianism would subsequently refuse to support antivivisectionist condemnation of all animal experimentation yet reserved the right to object to needless painful experimentation should it occur (AHA, 1892: pp. 31–33). This compromise allowed the humane movement to be united at a national level whilst providing enough flexibility to allow its constituent members to adopt whatever position on vivisection best suited local contexts and individual societies. In this way, humanitarians contained the danger that animal experimentation posed to the movement, allowing the AHA and its constituent membership to build a trusted reputation and credibility as custodians of American values, in contrast to the more radical antivivisectionist cause. In the years that followed, the AHA developed a conservative ethos marked by a reluctance to openly engage with the topic of animal experimentation, alongside antipathy toward the antivivisectionist movement. Pound release, therefore, posed a significant challenge to the AHA as it brought humanitarians and antivivisectionists together in common cause. Reflecting on the 1917 Californian experience, the *National Humane Review* warned that pound release risked “a war to a finish” setting the “forces of mercy and humanity” against science, medicine, and the state (Stillman, 1917a: p. 91). Whatever the outcome, it was difficult to see how the humanitarian cause would benefit from such a confrontation.

Antivivisectionists, however, recognized a clear advantage in continued battles against pound seizure as they might compel the humanitarian movement to publicly turn against animal experimentation. Indeed, one of the leading antivivisectionist journals, *The Starry Cross,* reported that pound seizure had proven “highly objectionable even to those who are not radically opposed to vivisection” as nobody could “look calmly on the possibility of his own dog being subjected to slow torture” (Nicholson, 1922: p. 170).

Antivivisectionists attempted to build upon their successful resistance to the pound release bill of 1917 by mounting a campaign to abolish vivisection in California. Harnessing a distinctive feature of Californian democracy, the optional referendum, petitions in 1920 and 1922 successfully triggered referenda but failed to gain traction with the voting public due to a vigorous defense of animal experimentation by the CSPMR.^5^ Whilst public and political concern could be mobilized against pound release, support for animal experimentation within the electorate remained robust. Consequently, subsequent legislative campaigns narrowed from state to local ordinances, adopting the language of humanitarianism as opposed to antivivisectionism and focusing on preventing the movement of animals from pound to laboratory rather than animal experimentation. On 27 September 1932, for instance, San Francisco passed a local ordinance establishing standards of care for the city pound. While explicit in having no intention to “prohibit vivisection in any way”, the ordinance prohibited the transfer of unclaimed animals to scientific research to “prevent exploitation of friendless, hopeless animals” (Anon, 1938b). With the passing of a similar ordinance in San Diego, a new and more subtle strategy cohered that used the specter of pound release to obtain legislation that would indirectly restrict animal experimentation. Elma Miller, committee member of the newly formed California Citizens’ Committee for State Humane Pound Legislation, explained that these local ordinances were to serve as a “model” for future legislative action as restrictive laws were a necessity “for safe-guarding dogs and their owners’ feelings” (Anon, 1938f). The California Citizens’ Committee for State Humane Pound Legislation aimed to extend local successes in San Francisco and San Diego to the entire state through a “uniform pound law” (CSPO, 1933: p. 3654; Anon, 1938e). The “State Humane Pound Act” proposed to regulate Californian pounds and was presented as a humanitarian intervention to protect vulnerable animals. The California Citizens’ Committee for State Humane Pound Legislation insisted that the law was not intended to restrict animal experimentation. Yet its provisions effectively prohibited the transfer of unclaimed animals to scientific research and its supporters were clear as to where the danger lay. Pet owning voters were warned that there was nothing to prevent “dog-bootleggers” colluding with poundmasters to sell lost animals to laboratories, and this could be “your dog, your faithful companion! And you could do nothing about it”.^6^

At the Forty-third Annual Meeting of the New England Anti-Vivisection Society, its president, George R. Farnum, had described the importance of focusing on the dog as a mechanism to achieve restrictive laws. Farnum explained that the “passage of an anti-vivisection measure in any part of the country would be an entering wedge” as there was “not a single line in any statute book of the United States … which even restricts animal experimentation” (Anon, 1938c p. 5). While Farnum was speaking of a bill to prohibit experiments on dogs in the District of Columbia, his words alarmed the CSPMR who suspected that the “State Humane Pound Act” was a “deliberate attempt to achieve antivivisection in California through the back door”**.**^7^ Members of the CSPMR began to warn of an “antivivisection measure under a new name—State Humane Pound Law” serving as:

an entering wedge for future and more drastic legislation. If it shall be adopted, not only freedom of medical research will be abolished and progress practically cease but without the continued use of animals the application of the knowledge thus far gained would be greatly curtailed (Coleman, 1938: p. 3).

The *Journal of the American Medical Association* took a broader view, arguing that the initiative “must be defeated” as the consequences were:

not confined to California. Just as this law is not primarily a humane pound law but an entering wedge for intolerable restrictions on the freedom of scientific research, so its adoption in California will be the signal for renewed campaigns in other States (Anon, 1938c: p. 582).

Days before bill went to a state-wide referendum on the 8th November 1938, the CSPMR contributed an article to the widely-read *Californian Monthly* exposing the humane pound bill as an attempt “to enact antivivisection legislation which the people and the legislature have already refused to accept, by concealing its real purpose under a cloak of sentiment”.^8^ The California Citizens’ Committee for State Humane Pound Legislation was “not exactly what it purported to be” as it was “adroitly camouflaged antivivisection” whose agenda was to “cripple medical research”.^9^ The officials of the California Citizens’ Committee for State Humane Pound Legislation were all shown to occupy leadership positions in the California State Antivivisection Society and the San Francisco Antivivisection Society. Through coordinated speeches, print media, radio, and widespread leafleting, the CSPMR delivered the message: “They CALL it the ‘State Humane Pound Law’ BUT it’s ANTIVIVISECTION all over again!”^10^ Informed of the duplicity, and warned of the threat to public health, the electorate rejected the bill in every county across the state with as much as 83.1% of the vote going against.

Whilst restrictive legislation had again failed to pass into law, the comprehensive defeat of the State Humane Pound Law was attributed to the covert antivivisectionist campaign lacking experience, political connections, and expert guidance. For instance, the antivivisectionist connections of the leaders of the California Citizens’ Committee for State Humane Pound Legislation was too transparent and their message inconsistent and confused. A number of blunders marred their campaign, such as acquiring and distributing thousands of leaflets that they believed had been issued by a sympathetic humanitarian society but which actually set out a robust defense of animal experimentation, criticized the needless waste of pound animals, and called for the rejection of the mislabeled “State humane Pound Law”.^11^ The leaflet had been produced by the CSPMR whose slick campaign against the bill, while slow to get off the ground, mobilized the full resources, connections, and influence of the medical profession to a spectacular success. Nevertheless, the rhetorical shift towards restricting the movement of animals to laboratories, rather than animal experimentation itself, had successfully attracted the support of the otherwise conservative humanitarian movement. Over fifty humanitarian societies and kennel clubs across California had campaigned in favor of the bill, alongside the State Humane Association and the wealthy Latham Foundation for the Promotion of Humane Education. Even the conservative leaning AHA, ever careful to avoid being embroiled in the antivivisectionist cause, had publicly supported the Californian State Humane Pound Law (Anon, 1938d). For antivivisectionists within California and for observers without, the movement of animals from pounds to laboratories appeared to have the potential to deliver legislation that would hinder animal experimentation, provided the political argument was properly cloaked as humanitarian reform.

## Normalizing pound release: medical science re-adopts an offensive strategy

Events in California were carefully watched across the nation. Maurice Visscher, a physiologist based at the University of Minnesota and vocal defender of animal experimentation, warned that if the American Medical Association (AMA) “does not fight this movement in California,” it risked being overwhelmed by antivivisectionist “cultists and racketeers”.^12^ Visscher’s concerns were widely shared, not least due to growing evidence of a nationwide strategy to pass antivivisectionist laws disguised as humanitarian reform.^13^ To counter this threat, the National Society for Medical Research (NSMR) was established to conduct nationwide public education on the benefits of animal experimentation, counter antivivisectionist propaganda, and support local efforts to combat campaigns for restrictive laws (see Parascandola, 2007). Almost immediately, however, NSMR strategy shifted as it recognized that “medical research loses public favor by appearing to oppose ‘humane’ proposals—no matter how fantastic these ‘humane’ proposals may actually be” (Carlson, 1947). Instead, the NSMR began to encourage grass-roots campaigns to obtain favorable animal research legislation with a focus on pound release. The aim was to counter the growing disruption to the supply of research animals caused by the antivivisectionist “new strategy”, which was beginning to frustrate medical researchers.^14^ When, for example, complaints of animal cruelty threatened the provision of dogs and cats to Johns Hopkins Medical School through animal-dealers, its Dean, Alan Chesney, declared a change in tactics now that the Maryland SPCA was “on the war path. I can assure you that our backs are up now, and that we propose to force the fighting.”^15^

This second wave of pound release campaigns initially emerged independently, responding to local disruption in cities and states across America. However, the NSMR worked to communicate strategy and best practice enabling distant medical communities to learn from others’ experience. The first significant success was the Michigan state pound release law, passed in on 19 June 1947. Led by Albert C. Furstenberg, dean of the University of Michigan Medical School, a broad coalition of medical interests sought to undermine antivivisectionism by passing a state law to regulate animal research. They passed this law in response to an attempt by antivivisectionists to impose upon the city of Detroit a legal injunction on the movement of animals from pounds to laboratories.^16^ In addition to pound release, the Michigan law required the licensing of laboratories, established a commission to regulate animal experimentation, and empowered State Commissioner of Health to assure compliance through inspection. *The Michigan Daily* characterized the campaign as “medical scientists … switching from the defensive to offense in supporting the first piece of positive animal experimentation legislation to come before the state legislature” (Anon, 1947a). The decision to focus on a state law stemmed from the belief that local campaigns for municipal ordinances often failed because they ran “afoul not only of the anti-vivisectionists but also the Societies for the Prevention of Cruelty to Animals, which are often merged with the former group or else have a great deal of local power”.^17^ Local humane societies were thought to be more willing to forge temporary alliances with antivivisectionists to counter pound release ordinances, even when some of their membership supported animal experimentation. In contrast, state societies were less agile and more cautious, in large part due to their broader constituency and lack of a direct responsibility for a pound or shelter. Like the AHA, state societies mainly served as the political representation of the movement which made it difficult to formulate a consistent public position on pound release let alone ally with antivivisectionist societies. In Michigan, for instance, the state-federated humane society “gave no trouble” on the condition that it be allowed to serve on the advisory commission established under the 1947 law to oversee humane standards of experimental animal care.^18^

By the late 1940s, a growing number of pound release campaigns were underway in cities (Albany, Baltimore, Buffalo, Cincinnati, Rochester) and states (Massachusetts, Pennsylvania, Minnesota, Nebraska, Wisconsin, Colorado, California) across America. Whilst the NSMR was careful not to instigate campaigns, it played an important role in helping to mold coherent and well-planned campaigns wherever they arose. George E. Wakerlin, Professor of Physiology at the University of Illinois School of Medicine, described the approach as “pyramiding”, where the NSMR ensured independent initiatives were networked and able to share strategy, experience, personnel, and materials (Wakerlin, 1947). The NSMR worked to change how medical scientists thought about their role by insisting they take responsibility for the public profile and legal status of animal research in their locality (Anon, 1949a, 1949b). Within a biological and medical community that was expanding rapidly through an influx of state and federal investment, the notion that scientists should play a public role was largely uncontested as science and state became increasingly interdependent. The National Institutes of Health (NIH), for example, issued federal guidance that encouraged the medical profession to engage local communities and government on the importance of a reliable dog supply for medical research. Furthermore, the NIH articulated an economic argument embedding unclaimed animals alongside scientific research as vital national resources that required rationalization. It would be:

a distinct waste of public money to impound dogs simply for destruction, and meanwhile to use additional public funds to purchase them for laboratories … [i]t is therefore essential that investigators work toward the adoption and maintenance of laws to prevent this waste (NIH, 1949: pp. 5-6).

In a later update to federal guidance, they designed a “model” state pound law, which the NIH encouraged all scientists to adapt and apply within their respective states (NIH, 1953: p. 7).

If local ordinances and state laws demanded the delivery of animals to laboratories, then pounds would either fall under the control of humane societies amenable to this new relationship with research institutions, and thus help repair relations between them, or, if entrenched in their objections to pound seizure, would have to give up running the municipal pound. Yet pound release laws did more than provide an inexpensive source of animals. From the perspective of Ralph Rohweder, executive secretary of the NSMR, cases such as Michigan demonstrated that medicine could ally with “constructive-minded humanitarians” to put an end to antivivisection by normalizing the movement of dogs from pounds to laboratories to benefit the public health.^19^ In doing so, the political argument as to whether animal experimentation was, or was not, humane was decided firmly in favor of medical science.

## Radicalizing the humane movement: American animal protectionism and the challenge of pound seizure

Rohweder’s optimism underestimated the significant challenge pound release posed to American humanitarianism. Within the AHA, it was well known that some pounds operated by humane societies made animals available to local laboratories. This could be undertaken openly, either informally or through a local ordinance, but more commonly went unpublicised. In 1949, for example, the Medical College of Alabama, Birmingham, had an understanding with the local pound that as “stray dogs” were killed “three times a week”, they could “call the day before requesting the number, sex and size of animals required [and] the manager of the pound saves those animals until our truck can pick them up” (Anon, 1949a: p. 2). The practice was so longstanding that nobody could remember how it came about. By the mid-1950s, however, local antivivisectionists had learned of this arrangement. The public controversy that ensued, forced the Medical College into a protracted legal battle to secure pound release legislation (Bolman, forthcoming). As pound release campaigns proliferated across the nation, the AHA struggled to foster a coherent response. Attentive to the varied approaches of constituent members, and aware of just how corrosive antivivisectionism could be to the national movement, the AHA had hitherto insisted that local societies decide for themselves how best to serve the humanitarian cause. This compromise was now untenable, not least because any ambiguity allowed critics to suggest that the AHA supported pound release. In 1947, as the organization struggled to develop a more coherent response, representatives of the AHA met secretly with those of the NSMR, agreeing to establish a joint committee to examine pound release. They hoped that by working together they could nullify antivivisectionism. Rohweder believed the collaboration was a “major step” that would “bring about in the minds of the public the proper division between the sane humane and dog breeders groups and the neurotic antivivisectionist groups”.^20^

The NSMR failed to appreciate the sensitivity of such a meeting, however, and its decision to report the development to its membership ignited a storm of controversy. Antivivisectionists and radical humanitarians accused the AHA leadership of conspiring with the “enemy” as they were in fear of a “coup d’etat” led by those willing to fight against pound seizure (Anon, 1947a, 1947b). Gustave Utke of the Wisconsin Humane Society, later explained to Rohweder the challenge posed by pound release:

I want you to know, confidentially, that I personally am not an Anti-Vivisectionist. However, there are things in the conduct of a humane society which, from the standpoint of commonsense, cannot be condoned. Perhaps of the most importance is that no animal should ever be given by a humane society to any laboratory for experimentation. It has been my feeling in the past that medical research laboratories, both local and national, have more or less maintained a hands-off attitude where humane societies are concerned. I believe you will find that most of the up-and-coming, humane societies, as well as the American Humane Association, are not in favor of the Anti-Vivisectionist cause one hundred per cent [but] It is also necessary for your group to understand … that we are supported through donation and that donations are made to our cause by people on both sides of the fence and to cause any discord on either side would mean a serious curtailment in our activities in the future.^21^

In response to the fallout from the NSMR meeting, the AHA president, Robert E. Sellar, was forced to declare that the AHA believed pound seizure legislation to be unconstitutional but took no position on animal experimentation. The *Christian Monitor* summarized the AHA position as being based on the rights of “private citizens… to organize within the law for a recognizable legitimate purpose with the assurance that this purpose will not later be perverted by the government” (Anon, 1952). As matter of civil liberty, the government had no right to compel organizations to act in a way that contradicted their legally established purposes. Moreover, legal status of property also seemed to make pound release unconstitutional. If animals were public property, movement to laboratories would contravene the principle that public resources should not be used to benefit private institutions. On the other hand, if animals were private property, then their movement from pound to the laboratory violated the US Constitution (which forbade deprivation of property without due process). By recasting pound seizure in terms of an unconstitutional exercise of power by government, the AHA hoped to avoid taking a position on animal experimentation. However, while politically coherent, this new tack did little to quell the growing controversy surrounding pound seizure within the AHA. It also proved to be misguided, setting the stage for a decade of chaos that served to radicalize and fragment the national humane movement.

In October 1950, the corrosive impact of pound seizure was revealed at the seventy-fourth AHA annual meeting when a group of objectors, latterly described as “antivivisectionists”, hijacked a presentation to demand action to prevent a pound seizure ordinance being passed by the city of Los Angeles (Anon, 1950a). Horace B. Sodt, then editor of the *National Humane Review,* hastily convened a special session to examine “dog seizure legislation”. The discussion was framed conservatively and concluded by endorsing the AHA position in the belief that the Los Angeles ordinance would be found unlawful. The controversy in Los Angeles had developed in 1949 when local antivivisectionists challenged the practices of the public pound, operated by the city, which was quietly releasing animals to medical laboratories. Confusion followed the public outcry, with the pound-master, Marvin Throndson, declaring that it was “not now and never has been the practice of the pound to surrender animals for vivisection”, while the Municipal Board of Animal Regulation quickly voted though a formal prohibition of the movement of animals from pound to laboratory (Anon, 1949b). A subsequent letter campaign, instigated by the recently formed Medical Research Society of Southern California (MRSSC), vehemently repudiated the decision (Anon, 1950b).^22^ The controversy escalated and resulted in the City Council being presented with two ordinances, one permissive (sponsored by the MRSSC) and one prohibitive (supported by local antivivisectionists). Following a “stormy” hearing, and a series of informal caucuses with much legal wrangling, both the Council and the Mayor were reluctant to endorse either ordinance. They sought a compromise in July 1950 by approving pound release while simultaneously voting to put the decision to the people as ‘proposition C’ in the forthcoming elections scheduled for November 7 (Anon, 1950c).^23^ While the AHA tried to resist being drawn into the affair, the State Humane Association of California vacillated, opposing “the surrender of animals from public animal shelters for any purpose which is not for the benefit of the animals released, including the purpose of experimentation”, but also emphasizing that “animal experimentation is and always has been quite lawful” (1950a). In the absence of a committed and forceful resistance from moderate humanitarians, and in the face of a well-organized medical campaign, the citizens of Los Angeles voted 357,393 to 261,699 in favor of pound release. It was at this point that the AHA leadership’s assumption that pound release laws would be found unlawful proved unfounded. The proceeding legal challenge to the Los Angeles ordinance was dismissed by the Californian Supreme Court. For Larry Andrews, this decision plunged the humane movement into “crisis” (Stiern, 1966: pp. 105–106).

The AHA’s leadership now came under increased and sustained attack by more radical members throughout the early 1950s. In 1950, the AHA leadership were embarrassed by an offer of $10,000 to fight back against the increasingly aggressive medical community. In 1952 conference members voted in favor of a proposition from the floor to censure any member of the society who willingly supplied animals to medical research. When the board of directors declined to act in both cases, this generated disquiet. A concerted effort was underway to reform and radicalize the AHA, and its leadership believed they were facing a determined effort by antivivisectionists to infiltrate and ultimately seize control of the wealthy and respected organization. Pound seizure was repeatedly used to portray the leadership as the “old guard” resistant to change and unable to defend the humane movement from attack. This was an effective strategy, aided by the democratic structure of the AHA. Historically, members were humane societies. Due to the geographic scale of the USA, a single representative of each society would often attend the annual meeting and vote on policies and programs on its behalf. However, the AHA constitution also allowed individuals to join as members and drew no distinction between the vote of a single individual and a vote on behalf of a member society. This made the Association vulnerable.

A significant shift in the direction of the AHA occurred in September 1951 when the little-known journalist Fred Myers was appointed to succeed the deceased Horace B. Sodt as editor of the *National Humane Review*. As the *National Humane Review* was the AHA’s primary means of communicating to members and wider public, Myer’s editorial stance would have easily been mistaken for a dramatic shift in the AHA’s position on antivivisection. The once cautious and conservative journal was now filled with regular reports on the dangers of pound seizure and aggressive attacks on medical research. Voices in support of pound release were suppressed.^24^ Through Myers, Larry Andrews was appointed director of the AHA’s field service. Both men worked to radicalize the AHA, building a small but effective group.^25^ This included members who were committed to, or sympathetic toward, the antivivisectionist cause such as Martha Flanagan and Robert Lindstrom, as well as others such as Christine Stevens who allied with Myers on the issue of pound seizure.

At the 1954 annual meeting, this group of reformers launched an attack on the leadership of the AHA from the floor. The leaders were accused of having knowingly constrained the progress of humane movement. Demands were made that the Association liquidate its near three-million-dollar endowment and spend the proceeds immediately on action against pound seizure. Attendance was unusually high and many of the interventions came from new members, some of whom had joined just before the meeting. They could easily outvote representatives of humane organizations. A central function of the annual meeting was to elect the leadership board. Conventionally, candidates were selected by a nominating committee and would then be ‘elected’ unopposed by attendees. But now an alternative slate was nominated from the floor with the promise to fight for the city pounds. A rancorous argument followed as radical proposals were countered, an attorney engaged to ensure due process, and what was conventionally a short process stretched to a 15-hour overnight marathon (Anon, 1954b). While the leadership successfully contained the more radical demands, it could do nothing to prevent alternative and member-nominated candidates being elected to fill three vacant seats on the fifteen-person board of directors. The first act of the newly elected directors was to request the resignation of any sitting director who refused to oppose pound seizure legislation.

By the time of the 1955 annual meeting, however, the more moderate members of the leadership board had taken several steps to re-establish their control. Known antivivisectionist members had been expelled, including Myers, who was stripped of his editorship of the *National Humane Review* and forbidden to attend annual meetings. With Myers removed, attacks on animal experimentation in the *National Humane Review* ceased. What appeared, at least to the outsiders, to have been an official and aggressive AHA campaign against pound seizure, ended abruptly without any formal explanation, much to the bewilderment of the NSMR. The AHA implemented a series of procedures to prevent further disruption from antivivisectionists, such as a ‘Committee on Resolutions’ to oversee AHA policies to prevent any surprise attacks from the floor. Constituent humane societies rallied in support of the leadership. Antivivisectionists were associated with anti-establishment politics and even accused of being part of a communist plot to subvert a leading custodian of American morality and, through attacks on medical science, undermine national security. When Martha Flanagan, Robert Lindstrom, and Christine Stevens stood as reform candidates in 1955, they found little support from a membership increasingly suspicious of their intent. The danger posed by pound seizure was seemingly contained.^26^

While the AHA reasserted itself as the respectable voice for animals within American society, the wider struggle for control of the humanitarian movement was far from over. Christine Stevens had already established her own independent humane organization in 1951, the Animal Welfare Institute (AWI). On failing to be elected to the board of the AHA, she set up the Society for Animal Protective Legislation (SAPL) in 1955. Myers, on departing the AHA, took with him Larry Andrews, Marcia Glaser, and Helen Jones, and established a rival and more radical organization: the National Humane Society, later renamed the Humane Society for the United States (HSUS). These new organizations did not have a large and broad membership. Their leaders were focused and strategic. Having witnessed the opportunity for coordinating local humane societies dissipate, and with it, the possibility of countering the well-synchronized campaigns of scientific and medical institutions, they would take the knowledge and skills developed over the many years of fighting over the local pound and target the federal government.

## The new humanitarianism and the laboratory animal welfare act

On the 24 August 1966, President Lyndon B. Johnson signed the Laboratory Animal Welfare Act “to end the business of stealing dogs and cats for sale to research facilities and to provide for humane handling and treatment of animals by dealers and research facilities” (Anon, 1966a). This was the first federal law passed that addressed the issue of animals in scientific research, and yet, its aim was to regulate the movement and care of animals destined for the laboratory, not their use in experimental practice. When Congressman Joseph Y. Resnick of New York opened the hearings, he was explicit in establishing that the bill H.R. 9743 was “concerned entirely with the theft of dogs and cats, and to a somewhat lesser degree, the indescribably filthy conditions in which they are kept by the dealer.” He remarked pointedly that he was “not an antivivisectionist and the issue of vivisection is nowhere involved in this legislation” (US House, 1965: p. 3). Resnick situated the need for federal intervention bill within the longstanding public and political concern over the movement of animals, specifically pets such as dogs and cats, across state lines for use in scientific research. “To the best of my knowledge”, he explained, “no dogs or cats are now raised for the laboratories. Under present conditions, a laboratory’s purchase order is an invitation for dealers to steal family pets. This is wrong … If dogs and cats are needed, as they most certainly are, let them be bred for the purpose’ (US House, 1965: pp. 4–5). While some of the most common laboratory species, such as mice and rats, were bred specifically for laboratory use, there was no pressing need to include them within the proposed law.^27^ The bill targeted the perceived growth in the illicit trade in stolen pets, long a concern of humanitarians and antivivisectionists, which was seen as a national problem.

In the decades following the close of the World War Two, the biomedical sciences expanded rapidly through huge government investment and the National Institutes of Health intramural and extramural research programs (Clarke et. al., 2010, Hannaway, 2008). In California, for example, biology and medicine had grown to become a significant component of the state’s research and educational system, and, in the 1960s, two further University of California medical schools opened at Davis and San Diego. This growth added to the impetus behind the renewed attempts to establish a state-wide pound release law in 1966. Robert N. Hamburger, assistant dean of the new School of Medicine of the University of California at San Diego, illustrated this broader scientific argument in arguing that medical access to pound animals would “hasten the end” of the dubious practice of “dealers” stealing animals to sell to laboratories (Libman, 1966). However, the Californian Senate Fact Finding Committee on the Supply of Dogs and Cats Used for Teaching and Research was not convinced by this argument. “Science”, the committee concluded, “requires uniformity in its experimental tools so that variables can be reduced…. Pound animals are arguably poor tools for scientific work because all of them are to some extent physically, temperamentally, and genetically unknown quantities (Stiern, 1967: p. 28). The Californian fact-finding committee had identified a prevailing trend that accompanied the rapid post-war expansion of the biomedical sciences. This was the concern that animals be purpose-bred, healthy, and well-cared for, to ensure consistent experimental results. While many saw the costs of breeding large animals such as dogs as prohibitive, random source animals (such as dogs from the pound) were increasingly viewed as unsuitable for much scientific work.

In 1949, and in the context of debates over the suitability of experimental animals and continued conflict over pound release, the medical establishment in California developed an alternative strategy. Unlike other legislative campaigns across America, what culminated in the California Animal Care Law (1952) made no mention of pound release. Instead, the aim was to enrol the government to improve “standards of animal care throughout the state and to ensure that inhuman practices or abnormal procedures do not exist” (Soave, 1954: p. 112). Medical schools, research institutions, and universities, coordinated by the California Medical Association, argued that the state was obliged to protect public investment in biomedical research. It was, after all, public money that was driving the rapid expansion of the biomedical sciences and placing intolerable pressure on an already stretched animal supply. There was nothing particularly unique in this argument. Similar trends were underway across the nation. However, for the medical profession to campaign on the single issue of state regulation of animal experimentation was unprecedented. Frustration was one significant explanation as to why state regulation of animal experimentation was deemed desirable. Decades of battles with humanitarians over pound animals was eroding public trust in science. For instance, even though institutions had established, and publicized, their own regulations governing the humane treatment of laboratory animals, this had done little to diminish the effect of antivivisectionist criticism on the public imagination. Moreover, there was no systematic approach to such policies that would enable local practices to advance and cohere into a shared community standard essential to the reliability of scientific knowledge. Government regulation, the California Medical Association hoped, would reassure the public while simultaneously benefitting science. But they would also ensure that scientists and medical experts, who appreciated the needs of researchers, would oversee the process. To this end, the Californian Animal Care Law made the State Department for Public Health responsible for the licensing and inspection of all (except federal) laboratories, while simultaneously establishing an Advisory Board, which consisted of members of the scientific and medical establishment and did not include any humanitarians, to oversee legal implementation and develop standards for the provision and treatment of laboratory animals.

However, the greater source of frustration remained the provision of animals. One unintended consequence of the frequent clashes over pound release was that the medical profession recognized how little was known about the sourcing of laboratory animals across California. At one university, for example, a dishonest dealer was found to have been purchasing excess animals from one department and selling them to the department next door at considerable profit for years without anybody noticing (Soave, 1954: p. 110). Accordingly, one of the first commitments undertaken by the Advisory Board was to construct a state-wide understanding of laboratory animal supply with a view to its rationalization and improvement. Taken as a whole, the Californian approach provided a “clearing house for information on laboratory animals”, enrolling state power to steer the improvement of the animal**-**dependent biomedical sciences by identifying, communicating, and encouraging best practice (Soave, 1954: p. 113–114). With moderate humanitarians and the wider public keen to see improved accountability, and politicians keen to resolve a longstanding controversy, the bill was passed swiftly in July 1952. While there were some fears expressed of the danger to scientific independence, most scientists thought the approach successfully elevated “standards of animal care not only through its police powers, but also through the interchange of ideas and methods throughout the state” (Soave, 1954: p. 115).

The California Medical Association’s move to embrace state regulation initially wrongfooted antivivisectionists and more radical humanitarians. Coinciding with the fracturing of the humanitarian movement, triggered in part by the failure to combat pound seizure in California, the California Animal Care Law immediately attracted the attention of a *new* humanitarianism. Liberated from the conservative politics of the AHA, organizations such as the HSUS and AWI sought to develop more aggressive and targeted strategies. Both were careful to distance themselves publicly from antivivisectionism, although representatives of the biomedical sciences remained unconvinced. In the 1950s, they began investigating the various uses of research animals. The AWI, led by Christine Stevens, adopted a more conciliatory approach, seeking to work with biological and medical researchers. The HSUS, under the guidance of Fred Myers, continued to fight against pound seizure and opposed any further expansion of animal experimentation (HSUS, 1965: p. 1). HSUS agents infiltrated laboratories to uncover the experience of animals used in scientific experimentation.

In 1958, Thomas Hammond, an undercover investigator, obtained the position of a laboratory technician in the animal surgery unit of the College of Medical Evangelists, White Memorial Hospital, Los Angeles. The HSUS targeted this laboratory because the city of Los Angeles had a pound release ordinance and California was the only state with dedicated legislation regulating animal research. Hammond’s reports of widespread animal cruelty were used by the HSUS to make a formal complaint under the Californian Animal Care Act (Anon, 1959). While an investigation by the State Board of Health found no violations, the issue generated significant public concern. It also once again focused attention on the supply of dogs, as the animals concerned had been obtained from the city pound (Anon, 1960a, 1960b).

In 1961, the HSUS embarked on a similar investigation of the shadowy network of animal dealers responsible for supplying dogs and cats to laboratories across the nation. Frank J. McMahon, HSUS Director of Field Services, compiled compelling evidence that demonstrated how state anti-cruelty laws were inadequate in controlling the booming trade in animals. There were numerous examples of cruelty and unhygienic housing in the collection and transportation of animals. The most inflammatory findings suggested that pet theft was routine and municipal pounds were complicit in the practice. By developing techniques such as the careful recording of the coat patterns of lost animals, McMahon became an expert in tracing pets trafficked across state lines from homes via dealers and pounds to laboratories. In 1965, for example, McMahon traced the movement of a missing pet dog named Teenie from Boyce (Virginia) to facilities of the National Institutes of Health (NIH) in Poolesville, Maryland, through a large east coast laboratory animal supplier, Lone Trail Kennels, based in Pennsylvania (Anon, 1965). Stolen animals were stripped of all identification and moved across state lines to escape local jurisdiction (US House, 1966: p. 3). The problem was a national one and so beyond the power of city, county, and state governments to police, and the HSUS was building a case for federal intervention.

On the 4 February 1966, the “lucrative and unsavory” trade in pet animals was exposed as a national scandal when *Life* magazine covered in disturbing detail so-called “concentration camps for dogs” (Wayman, 1966). Christine Stevens had convinced the magazine’s owner, Henry Luce, to publicize the trade in animals, leading to a photographer, Stan Wayman, accompanying McMahon as he investigated a Maryland dog dealer named Lester Brown (Stevens, 1974). Wayman’s photographic account of malnourished and fearful dogs kept in abhorrent conditions showed how, in his words, “unscrupulous dog dealers” were profiting from the demand for animals in medical research by providing “pets for sale—no questions asked” (Wayman, 1966: p. 22).

By 1960, we see the new humanitarian organizations fighting for a federal law as the best mechanism to improve the scientific treatment of laboratory animals. Christine Stevens’s SAPL coordinated the introduction of parallel bills to address laboratory animal welfare in the Senate and House. The HSUS, however, objected on the grounds that they made the Department of Health Education and Welfare responsible for policing animal experimentation. Recent experience in California had demonstrated the danger of self-regulation. Accordingly, in 1961, the HSUS endorsed a bill to create a new and independent regulatory authority, the Agency of Laboratory Animal Control, located within the Department of Justice (US House 1962). However, both these attempts were rejected by the National Anti-Vivisection Society who wished to abolish, not regulate, animal experimentation, placing them in a paradoxical alliance with the NSMR (Stevens, 1990: p. 72). Given these divisions among animal protectionists, the united biomedical community easily ensured that the proposal for federal regulation of animal experimentation was lost in committee.

By the mid-1960s, the AWI and HSUS strategy had shifted and returned to the issue with which they had experienced more success – the movement of animals to laboratories. The graphic coverage by *Life* magazine, together with an earlier detailed account of “dognapping” carried by *Sports Illustrated* (another popular Luce title), focused public and political attention as never before on a national problem that struck at the heart of American society. Resnick had been moved to introduce a bill in 1965 to prevent “dognapping” in response to the story of a stolen pet dalmatian named Pepper, stolen from her home in Slatington, Pennsylvania, and surreptitiously trafficked to New York City’s Montefiore Hospital, where she had been experimented on, killed, and cremated. When *Sports Illustrated* broke the story it warned of the need to protect American pets from “unscrupulous and cruel professional dognappers” because:

almost as much as a child, the domestic dog is part of the human heart and the human home and has been since lost time, for reasons no one can or need explain. Although the love men have for dogs is a socially accepted fact, it is one that at present does not have much legal recognition (Phinizy, 1965: p. 42).

*Life* magazine similarly presented “dognapping” as national in scope and a threat to the American family as “pets, trained to be obedience and easy to handle, are especially prized, and the Humane Society of the U.S. estimate that 50% of all missing pets have been stolen by ‘dognappers’ who in turn sell them to dealers” (Wayman, 1966: p. 22).

Supported by such media coverage, the AWI and the HSUS pressed for action to curtail dognapping and regulate animal dealers and a flurry of bills designed “to regulate the transportation, sale, and handling of dogs and cats” were debated from September 1965 through to the summer of 1966. Appearing before the House Subcommittee on Livestock and Feed Grains of the Committee on Agriculture, McMahon spoke of the prevalence of cruelty practiced by animal dealers and the tremendous scale of their practice, extending to the “corruption of humane society employees and public employees” (US House, 1965: p. 25). In one case, the HSUS was asked to take over the running of the municipal pound in Camden, New Jersey, having revealed that its employees were “selling animals to commercial laboratory suppliers on the same day they were received at the pound, making it impossible for owners to reclaim a lost or strayed pet” (US House, 1965: p. 25).

The need to regulate commercial dealing in animals dovetailed with the longstanding critique of the movement of animals from pounds to laboratories and provided grounds for an explicit counterargument to pound release laws operating across the nation. Christine Stevens, for example, presented the “theft and mistreatment of dogs and cats for sale to laboratories” as a “wide-spread problem of long standing” that could not be disentangled from the issue of pound seizure (US House, 1965: p. 37). Rather than a solution to the problem, the movement of animals from pounds to laboratories was an integral component of the illegal circulation of animals. Stevens argued that, in the eleven states and thirty municipalities known to have pound seizure laws, animal cruelty and pet theft were rife. After all, she contended, “New York has such a law, yet the very incident which caused Congressman Resnick to decide to introduce legislation on this subject relates to a Pennsylvania dog, transported by a Pennsylvania dog dealer to a laboratory in New York City” (US House, 1965: p. 40). Stevens went further, however, not content with legislation to control animal dealers, and supported only those bills that established the right of the Secretary of State to regulate standards of care *within* laboratories (US House, 1965: p. 40).

The biomedical community was caught between their longstanding resistance to external regulation and their recognition that something needed to be done to improve the supply of animals and quell growing public concern. Visscher, now president of the NSMR, spoke clearly and consistency against the need for legislation that targeted biomedical research. Visscher had an imposing reputation, having campaigned against antivivisection for decades. His testimony reflected his long experience of countering legislation to prevent animal cruelty that seemed designed to cripple medical experimentation. He saw in the legislation governing the movement of animals another veiled attempt by antivivisectionists to curb biomedical access to animals. Visscher dismissed forcefully and at length any need to regulate experimental practice, arguing that the recently established American Association for Accreditation of Animal Laboratory Care ensured “proper housing and care for animals in all scientific laboratories.” In any case, the Department of Health, Education, and Welfare was preparing its own bill on this issue. As to pet theft, the solution was simple and well-established, as all states “should have laws making it mandatory for public pounds and any other animal shelter operating under police authority, to give animals which would otherwise be uselessly destroyed to appropriate institutions for use in scientific study” (US House, 1966a: pp. 46–47). This would cripple the trade in stolen animals. He also highlighted contradictions. The bills were wholly focused on the stealing of animals for laboratories, and ignored theft for other purposes, while attempts to expand the bills to include other small animals such as rats and mice made no sense as they were not at risk of theft. Walter Brooker of Howard University College of Medicine made the point more directly, suggesting the real interest was “to make more difficult the use of animals in teaching and in research” and should members of the committee doubt this” then all they need do is:

consider the activities of many of the people who favor this bill… What was their position in the 1940’s and 1950’s in Chicago, in Boston, in Buffalo when the cities developed programs to turn over to medical schools all unclaimed animals in the pounds rather than to put them to death serving no purpose? Ask them if they opposed and subsequently did everything they could do to obstruct such an arrangement (US House, 1965: p. 30).

The argument for pound release as a solution to pet theft had changed little since originally presented in the opening decades of the twentieth century (cf. Whipple, 1917). William Putney of the Medical Research Association of California testified that since the Los Angeles pound release ordinance of 1951 there had been “no dognapping for the purpose of sale to medical research institutions” in the city (US House, 1966a: p. 182). Bernard Baltes, a Californian biomedical researcher, was “astonished” by the call for legislation to curb animal dealers as the antivivisectionists and humanitarians:

in all their righteousness, have actually created this monster, ’the dognaper’, by influencing individuals to destroy animals rather than release them for research and experimentation. Then on top of this they ask the Federal Government to destroy the monster by strict regulations when all could be handled so easily as we have done in California where the animals are made available to us [via pound release] (US House, 1966a: p. 185).

Oscar Sussman, a veterinarian at the New Jersey State Department of Health, concurred, comparing restrictions against pound release to the impact of prohibition on alcohol consumption in “fostering the very thing that this committee is trying to eliminate” by “causing the price of animals to go up and allowing these unscrupulously filthy-minded [animal dealers] to make money from the sad situation” (US House, 1966a: p. 159). Numerous biomedical witnesses urged members of Congress to study California and the ongoing process to establish a state law compelling the release of impounded animals. Betty Shapiro, chair of the Los Angeles County Health Commission, summarized these arguments with the simple statement that “[ever]y state should have such a law” (US House, 1966a: p. 185). As coherent and compelling as these arguments may have been, they possessed one significant flaw. As we have seen, the Californian Senate Fact Finding Committee on Public Health and Safety was reaching the conclusion that a pound release law would not serve the interests of biomedical research.

The foundation of the Animal Care Panel in 1950 by a group of veterinarians working in research laboratories, the publication of the first edition of the canonical *Guide for Laboratory Animal Facilities and Care* (ACP, 1963), and the formation of American Association for Accreditation of Animal Laboratory Care in 1965, indicated that the expansion of the biomedical sciences had not only generated a greater demand for animals, but an interest in their origins, health, and quality. As Visscher had testified, the fact that the DHEW was preparing its own bill on federal regulation on the welfare of animals, meant that the bills addressing pet theft would risk duplicating responsibility across federal departments if they were to include laboratories within their scope. He was supported in his argument by Helen B. Taussig, a Johns Hopkins cardiologist appearing before the hearings on behalf of the American Heart Association. Taussig was renowned for having created, with Alfred Blalock and Vivien Thomas, a method for surgically correcting a congenital heart defect responsible for ‘blue baby’ syndrome through extensive experimentation with dogs (US House, 1966a: p. 70). Taussig explained that to improve animal welfare “medical institutes need money for renovation and reconstruction of the animal quarters.” Yet when their funding requests were assessed by federal bodies, it was these “requests for better animal quarters” that were “likely to be knocked off the bill” (US House, 1966a: p. 70). Taussig described the sourcing of laboratory animals as experiencing a moment of transition, moving from a chaotic and underfunded patchwork of approaches to a more purposeful system prioritising the health, welfare, and overall suitability of animals for experimental research. Until that transition was achieved, the fault was not with the laboratory. The “mishandling of animals really comes in the source from which animals are procured” for which, at least in the short-term, “good permissive pound laws are the answer” (US House, 1966a: p. 70). For humanitarians, however, it was this increased focus on the suitability of animals that was driving the surge in pet theft. Jacques Sichel of the HSUS agreed that “laboratories do not want… strays picked up off the streets” as they were “derelicts … starved, diseased, and old… not fit subjects for scientific research.” But this meant that the demand for healthy animals was “met by illicit means” (US House, 1966a: p. 194).

Within the House of Representatives, it was the arguments of scientists and medical experts that proved persuasive; on the 29 April 1966 an amended version of a bill passed, limiting protection to dogs and cats and, very controversially, weakening controls over animal dealers and removing research institutions entirely from the scope of regulation. *The Washington Post*, reflecting the public mood, condemned this watering down and called for “stronger legislation”. Within the Senate, however, a further amendment led to more hearings and the passing of a strengthened bill. Despite urging from many humanitarians, not least Christine Stevens, Senate legislators refused to include the practice of animal experimentation. But neither were they willing to focus their attention on dealers alone. A middle way was required:

it is not just the animal on the way to the laboratory that is faced with inadequate care and treatment. The committee hearings disclosed that shortcomings existed in the care and housing that animals receive after arriving in many medical research laboratories. Cramped quarters and inadequate care are often present, especially in the older research institutions. H.R. 13881 as amended by the committee also recognizes the need for upgrading animal standards in the laboratory, but at the same time provides adequate safeguards to insure that medical research will not be impaired (US Senate, 1966b: p. 5).

This compromise was a solution to the political problem faced by legislators as opposed to the practical problem experienced by biomedical and humanitarian interests concerned with laboratory animal supply. For instance, recommending that the Department of Agriculture take on responsibility for regulating the handling and transport of laboratory animals was a political resolution of a vexed question that required an extra day of hearings to reach. It made little sense to the DHEW, which possessed relevant expertise on laboratory animals, or the Department of Agriculture, which did not. The biomedical community, which wanted to exclude research institutions from regulatory scope and focus on animal dealers, argued that the DHEW was the accepted authority and most capable of improving the supply of laboratory animals. Among the humanitarians, Christine Stevens and the AWI were comfortable with the DHEW acting as the authority (particularly if regulation extended into the laboratory). However, the HSUS, with their recent experience of the Californian Animal Care law in mind, rejected outright any suggestion that laboratory animal welfare be regulated by an authority with vested interest in the biomedical research. Conflicting bills passed by Senate and House necessitated a conference to produce an effective compromise by selecting and combining “most practicable provisions of each version … in an effort to produce workable and meaningful legislation” (US House, 1966c: p. 6). The settlement, which incorporated the general ethos of the Senate committee’s recommendations, passed through both houses, and was signed into law as the Laboratory Animal Welfare Act (1966) by President Johnson on 16 August 1966.^28^

## Conclusion

The movement of animals, particularly dogs, across the spaces of the home, city street, local pound, dealer’s yard, and medical research laboratory, has been subject to intense and vitriolic contestation throughout the twentieth century. There have been countless ordinances, numerous bills, and even so-called dog referendums in American cities and states. We have explored how the circulation of animals from streets and homes to pounds and then, most controversially, from pounds and dealers to medical institutions, was of critical importance to three communities, broadly defined. For antivivisectionist activists, the pound was a means of saving animals and starving the medical institutions of experimental subjects. But it was also a strategy to ally their politics with the more established, influential, and credible humane movement. Some in the humane movement reciprocated as the defense of the shelter and the appeal to family values through valorization of the pet forged a common concern. Together they transformed American humanitarianism. For the biological and medical community, pound release was more than a pragmatic and economic tool to obtain animals for experimental research. Ordinances and state law established a social contract in law for animal research, undermining the criticisms of antivivisectionists, preventing their attempts to disrupt and block the movement of animals to laboratories, and denying them an important source of power, capital, and influence in local communities. In organizing at local levels to campaign for permissive laws, the role of the medical professional was expanded to include obligation to societal engagement and public education.

In this essay we have worked to locate the Laboratory Animal Welfare Act of 1966 within a context of this long and protracted debate over the movement and place of animals in American society. In so doing, we have sought to address what Novak (2008) has recognized as the productive potential of the “sprawling disarray” of state power and civil society on the periphery. Understanding American state power as dispersed and horizontally organized, rather than vertically consolidated, is of direct consequence to the development of the biomedical sciences, and to the history of animal experimentation, the politics of animal welfare, and the legal status of non-human organisms, where scholarship has flourished in recent decades. When we look to the history of antivivisectionism, we see it attending to two periods (Lederer, 1992; Parascandola, 2007; Ross, 2014; Rudacille, 2000; Vetri, 1987). The first is when antivivisectionists commanded an impressive amount of public support in late nineteenth and early twentieth centuries. The second period is in the 1960s when there was legal wrangling in Washington stimulated by the undercover investigations of cruelty in animal trade. When historians and legal scholars have looked at the issue of the pound, they have focused on the post-war era, particularly the 1950s and 60 s. The analysis has been largely limited to the philosophy and tactics of the major national bodies, such as the NSMR and AWI, and the federal legislation that is seen as resulting from this struggle.

Consequently, the long history of the intense and persistent fight over the pound across the United States has not been recognized or, if recognized, treated superficially. When scholars identify how the tragic tale of Pepper the dalmatian, stolen from a front yard in Pennsylvania and dying in an experimental procedure in New York in spite of her family’s frantic searching, generated national media attention and spurred political action in Washington, or when they note how the *Life Magazine* story of mistreated dogs “led to a grassroots uprising in which voters bombarded Congress with outrage”, they fail to recognize how these were well-established tactics with a long history (Phillips & Bellotti, 2017). With these two events, activists had not, as Jordan Curnutt (2001) has argued, “stumbled upon the strategy that would prove most effective for establishing the legal regulation of care and use of research animals”. Rather, this was the strategy which had been developed and refined through decades of confrontation between local humane and anti-vivisectionist activists and the hospitals and universities in towns and cities across America. Likewise, when the scientific and medical establishment countered in the hearings of 1965 and 1966, they turned their attention to the issue of pound release, a subject where they had refined their modes of argumentation and begun to score some notable legislative successes.

In their campaigns to protect lost and stolen pets from being trafficked to laboratories, twentieth-century antivivisectionists had developed a powerful technique that helped build alliances and appeared capable of mobilizing public and political opinion. However, having allied their cause with the sanctity of the animal shelter, antivivisectionists struggled to capitalize on legislative momentum to pass restrictive laws on the practice of animal experimentation itself. Rather than focusing on what happened to animals within the laboratory, public and political concern limited itself to the movement of animals and the question of which animals belonged in which societal spaces. Antivivisectionists and humanitarians saw the proper place of the dog and cat to be that of the family home. Outside of it, their lives were characterized by sickness, pain, and suffering. Hence the critical role of the shelter in finding homes for the lost and ownerless animal or, failing this, in providing a painless and humane death. The dog possessed a purpose and a life worth living only as part of the human family. In contrast, the biomedical community (as well as some humanitarians), viewed the unclaimed and homeless dog as a valuable research tool that could be used to advance the public health. Public and political opinion spanned these perspectives, producing a compromise that limited federal oversight to animals that had the potential to have been moved out of their appropriate societal context. Species such as mice, for instance, now purpose bred for scientific use, were in their appropriate place when in the laboratory and therefore obtained little to no privileged legal protection under the Laboratory Animal Welfare Act (1966). When an animal was *out of place* there was problem, but otherwise there was not. Dogs and cats when in the laboratory were potentially animals out of their proper societal place as they had other cultural roles to play, roles that for many were deemed more significant and socially valuable than that of a laboratory animal.^29^ As such, the focus of concern was the breeding, care, and transport of animals prior to their entry to the laboratory. The Laboratory Animal Welfare Act (1966) regulated the movement and care of animals to the threshold of the experimental laboratory and no further.

## Footnotes

1 Wang draws from Novak (2008: p. 766) who argues that looking at the “state in action rather than in theory” reveals that “looking simply at the national center or the federal bureaucracy is to miss where much of the action is—at the local and state levels—on the periphery”.

2 Public Law 98–544 was commonly known as the ‘Laboratory Animal Welfare Act’ until an amendment in 1970 formalized its name as the ‘Animal Welfare Act’ (US Public Law 91–579 1970). The 1970 amendments expanded the number of species covered by the law and extended the definition of ‘dealer’ to include premises that predominantly traded in pet animals which also sold animals for research. More significantly, it expanded the scope of regulation to encompass animal experimentation directly by mandating all registered research laboratories demonstrate the appropriate use of anesthetic, analgesic, and tranquilizing drugs… during actual research or experimentation” (US Public Law 91–579). For an extensive study of how regulations developed in the 1980s, see Carbone (2004).

3 CSPMR, Campaign to Defeat the Proposed “State Humane Pound Act” Initiative in California, November 8th 1938, Final Report. In papers of the California Society for the Promotion of Medical Research, MSS 71–2, (hereafter CSPMR Papers), University of California, San Francisco, Carton 2.

4 Contractual arrangements varied widely. In smaller towns it was common for a fixed appropriation to cover expenses, while in cities the power to administer dog licenses and retain fees collected to cover the cost of animal control was frequently delegated to humane societies (Shultz, 1924: p. 95).

5 The optional referendum allowed voters to propose and pass their own statutes and constitutional amendments subject to reaching a set threshold of support to trigger a state-wide vote. It was introduced as part of a suite of progressive political reforms in 1911, which included widening the franchise to include women (a move that likely expanded the antivivisectionist vote).

6 A. L. Rosemont, editor of Western Kennel World, and Vincent J. Garrity, “Argument in Favor of Initiative Proposition No. 2, CSPMR Papers, Carton 2.

7 S. P. Lucia, “Unmasking “The State Humane Pound Act”, draft for *California Monthly* 8 September 1938, p. 5, CSPMR Papers, Carton 2. The antivivisectionist language of “entering wedge” used in the context of the District of Columbia campaign equally alarmed medical scientists across the East Coast not least George H. Whipple who had relocated to Rochester School of Medicine in the intervening period (Lederer 1992: p. 65).

8 Robert Gordon Sproul, President of University of California, quoted in Lucia, “Unmasking”, ibid., p. 12.

9 Campaign to Defeat the Proposed “State Humane Pound Act”, ibid.

10 CSPMR, Campaign to Defeat, ibid., p. 2. See also Anon (1938 h).

11 CSPMR, Campaign to Defeat, ibid., p. 40.

12 M. Visscher, letter to B. O. Raulston (University of Southern California), 24 March 1938. CSPMR Papers, Carton 2, Folder: Speeches, statements, articles, et cetera.

13 For instance, the New England Anti-Vivisectionist Society was found to have “given generous financial aid to the Humane Pound Bill in California”. CSPMR, Release, 2 November 1938. CSPMR Papers, Carton 2.

14 Campaign to Defeat the Proposed “State Humane Pound Act”, ibid.

15 Chesney to Carlson, Dec 2, 1948, ibid.

16 In Detroit “worthy and valuable” unclaimed dogs could be sold at auction, a convention that local antivivisectionists challenged on the grounds that dogs were only “worthy and valuable” when they were pets (Kemp, 1948).

17 Lovell, R., letter to A. C. Barger (Harvard University), 10 January 1957. National Society for Medical Research Archives, MS C 417, Modern Manuscripts Collection, History of Medicine Division, National Library of Medicine, Bethesda, MD, (hereafter NSMR Archives), Box 19, Folder ‘Pound Laws, Michigan”.

18 Kemp, H. A., letter to A. J. Carlson, 14 July 1948. NSMR Archives, Box 19, Folder ‘Pound Laws, Michigan”.

19 R. A. Rohweder, letter to W. A. Young, 27 June 1951. NSMR Archives, Box 11, File: “Humane Leaders”.

20 R. A. Rohweder, Meetings of the Board of Directors, 9 February 1947. NSMR Archives, Box 3, File: Memos 1949–1960.

21 G. Utke, letter to R. A. Rohweder, 9 March 1949, NSMR Archives, Box 11, File: “Humane Leaders”.

22 The MRSSC was established in 1948 to “further high standards of medical research and education and to foster public interest in the need of research” (Anon, 1948). Like the CSPMR, the impetus was a response to antivivisectionist attempts to prohibit access to research animals (Anon, 1950e).

23 Faced with two conflicting requests, Los Angeles Mayor Fletcher Bowron shirked the challenge by refusing to sign the permissive ordinance endorsing only the decision to put the question to the people. The city’s failure to demonstrate leadership attracted widespread condemnation, with the *Los Angeles Times* declaring it “weak, if not dangerous” having “not only put the cart before the horse they put the horse in the cart and passed it to the public to get him out and on the road again” (Anon, 1950d).

24 Albert H. MacCarthy, President of the SPCA of Anne Arundel County, found the *National Humane Review* resolute in its refusal to acknowledge, let alone publish, his societies objection to any move to compel members to oppose pound release. MacCarthy, Open Letter: The Society for the Prevention of Cruelty to Animals, Anne Arundel County, Maryland, 5 October 1934, Alan Mason Chesney Papers, Alan Mason Chesney Medical Archives of the Johns Hopkins Medical Institutions, Baltimore, Maryland, Box 6, Folder: Correspondence, Maryland Society for Medical Research, 1954–1958.

25 Whether or not Myers was committed to antivivisectionism prior to his appointment, there is scant evidence. While Andrews had a background in the humane and animal protection movement, Myers did not. Myers had worked for numerous local papers before becoming an organiser for The News Guild, a union for communication workers of America, also acting as editor of *The Guild Reporter*.

26 Myers repeatedly denied being a communist though was widely thought to be a “fellow traveller”. Having served as executive secretary of the American-Russian Institute, viewed at the time as a subversive organization, in 1956 he was brought before the Eastland Committee examining the scope of Soviet Activity. Stevens, in contrast, was the wife of Roger L. Stevens a New York property developer with strong connections within the democratic party. This gave Stevens considerable political influence enabling her to enrol congressmen and women to her cause in ways that other animal advocates could not. Despite Myers and Stevens very different political positions, the two could work together when their interests aligned such as in their attempt to reform the AHA.

27 Which species were to included, and how legislation would apply to a species if included, was one of the most contested issues throughout he hearings and congressional debate, only being resolved during a committee of conference which met to reconcile differences between U.S. Senate and House. The outcome, which saw the Laboratory Animal Welfare Act (1966) include monkeys, guinea pigs, hamsters, and rabbits, as well as cats and dogs, although provisions differed across these species, was a political compromise balancing humane concern against economic and practical considerations (see US House, 1966c). While we have not explicitly made the argument here, we do suggest that this wider context may have encouraged the development of purpose bred laboratory dogs (for the latter, see Bolman, 2021).

28 In 1966, the Californian Animal Care Law (1952) and federal Laboratory Animal Welfare Act (1966) addressed different aspects of the scientific use of animals. For example, the California state law regulated the treatment of animals during experimentation whereas federal legislation explicitly excluded the experimental use of animals from its provisions. Only federal law regulated dog and cat dealers. While both laws regulated the care of animals before and after experimental use, they differed considerably in approach. For example, federal law covered monkeys, guinea-pigs, hamsters, and rabbits in laboratories that also used dogs and cats, whereas Californian state law included all species. Furthermore, Californian state law excluded federal laboratories whereas federal law included them. As such, in California, state and federal law could co-exist and continued to do so as federal law gradually expanded its scope. At the time of writing, state regulation of the use of laboratory continues to form part of the California Code of Regulations. Title 17. Public Health. Division 1. State Department of Health Services. Chapter 2. Laboratories. Subchapter 1. Service Laboratories. Group 5. Care of Laboratory Animals (17 CCR §§ 1150−1159).

29 As a result of the final conference to bring consensus between the Senate and House, monkeys, guinea-pigs, hamsters, and rabbits were partially protected in a compromise that saw their inclusion within standards of care as obligatory, but records of their identity, purchase, sale and transportation were not required as they were as they were not at risk of ‘pet’ theft (US House, 1966c).
